# Pyrolysis-Dependent Uncollided Neutron Transmission in Date-Palm Biochar/Epoxy Composites: A GEANT4 Study with 2 MeV and Cf-252 Neutrons

**DOI:** 10.3390/polym18212687

**Published:** 2026-10-29

**Authors:** Ahmed Alharbi

**Affiliations:** Department of Physics, College of Science, Qassim University, Buraydah 51452, Qassim, Saudi Arabia; ahma.alharbi@qu.edu.sa

**Keywords:** biochar, date-palm waste, epoxy composite, neutron shielding, uncollided transmission, neutron fluence, ambient dose equivalent, ^252^Cf, GEANT4, biomass valorization

## Abstract

Pyrolysis changes both the composition and physical structure of biochar, making it necessary to separate chemistry-driven neutron-transport effects from density and geometry effects. This computational study examines date-palm biochars produced at 300, 500, and 800 °C as fillers in an idealized epoxy matrix. The principal 2 MeV comparison used ten independent $10^{6}$-history GEANT4 runs per material at 20 wt% filler, 5 cm thickness, and equal bulk density (1.25 g cm^−3^). From BC300 to BC800, total forward track-length fluence increased by only 0.30%, whereas the fluence-weighted $H^{*}\left(10\right)$ response increased by 2.86%. A separate ten-replicate component campaign confirmed that the 2 MeV BC800–BC300 $H^{*}\left(10\right)$ difference (+0.07986 pSv primary^−1^) was driven by increased uncollided-primary transmission (+0.08398 pSv primary^−1^), while the collided-primary contribution decreased slightly (−0.00422 pSv primary^−1^). For a representative ^252^Cf Watt spectrum, $H^{*}\left(10\right)$ increased by 2.98%, with 91.1% of the net increase arising from uncollided primaries. A 30×30 cm^2^ full-face geometry increased scattered buildup, yet 84.0% of the BC800–BC300 $H^{*}\left(10\right)$ increase remained attributable to uncollided primaries. Additional density, thickness, and loading sensitivities gave positive BC800–BC300 $H^{*}\left(10\right)$ contrasts under all tested conditions (1.18–8.24%), although the integral-fluence contrast was not universally positive. The model therefore supports a robust chemistry-related increase in high-energy primary survival while showing that the scattered-field partition and engineering performance remain geometry- and processing-dependent.

## 1. Introduction

Biochar is a carbon-rich solid produced by thermochemical conversion of biomass under oxygen-limited conditions. Its use as a polymer-composite filler is attractive because it couples biomass valorization with changes in stiffness, thermal stability, electrical response, rheology, and other functional properties [1,2,3,4]. These properties are not intrinsic to a single “biochar” material: feedstock, pyrolysis temperature, residence time, particle size, surface chemistry, pore structure, and post-processing all affect the resulting filler and its interaction with a polymer matrix [1,3,4]. Epoxy/biochar systems similarly show formulation-dependent behavior, so a radiation-shielding assessment should not treat biochar as a chemically or structurally invariant additive [5].

For neutron shielding, this variability is particularly important because fast-neutron moderation depends strongly on hydrogen atom density and on the probability of elastic scattering, while absorption and additional scattering depend on the elemental and isotopic composition of the full formulation. Hydrogen-rich polymers are therefore widely used as neutron moderators, frequently together with boron-containing phases that enhance absorption after moderation. Experimental and computational studies of polyethylene/B_4_C and epoxy/UHMWPE/B_4_C systems illustrate the coupled roles of hydrogen content, absorber loading, density, and microstructure in practical neutron-shielding composites [6,7,8]. Such studies also show why shielding performance cannot be inferred from filler identity alone.

Biochar has recently been considered as a lower-cost, biomass-derived shielding additive. Martellucci and Torsello used PHITS to investigate biochar-reinforced concrete and showed that attenuation depends on both biochar fraction and composite density [9]. That work established the relevance of biochar chemistry to neutron transport, but it did not isolate how pyrolysis-dependent composition changes the balance between uncollided and scattered neutrons in a polymer matrix. This distinction matters because a change in total transmitted fluence can be small even when the energy distribution, and therefore a fluence-to-dose-weighted quantity such as $H^{*}\left(10\right)$, changes appreciably.

Date-palm residues provide a useful controlled case because Usman et al. reported substantial composition changes between 300 and 800 °C [10]. Carbon increases with charring temperature, whereas hydrogen and oxygen decrease and ash becomes more concentrated. At the same time, real pyrolysis also changes particle density, bulk density, porosity, surface area, particle structure, moisture affinity, and interfacial behavior [3,4,11]. Consequently, an equal-density calculation can isolate the chemistry contribution but cannot by itself predict the behavior of fabricated composites.

The novelty of the present study is therefore not a generic demonstration that hydrogen-rich materials moderate neutrons. Rather, it quantifies how measured pyrolysis-dependent date-palm composition changes are partitioned among uncollided-primary, collided-primary, and secondary-neutron contributions in an epoxy matrix, tests whether the mechanism persists for a representative ^252^Cf fission spectrum, and evaluates its sensitivity to density, thickness, filler loading, and a substantially larger full-face geometry. The intended application is computational screening of polymer formulations before fabrication for radiation-protection components, including laboratory, nuclear-technology, accelerator, medical, and other environments where lightweight multifunctional shielding materials may be considered. The calculations remain model predictions and do not replace experimental characterization and transmission measurements.

## 2. Materials and Methods

### 2.1. Date-Palm Biochar Composition

The modeled fillers correspond to date-palm biochars prepared at 300, 500, and 800 °C (BC300, BC500, and BC800) in the study of Usman et al. [10]. The principal elemental inputs are listed in Table 1. The Ca, Mg, K, and Na concentrations reported for the biochars were treated as constituents within the measured ash fraction, not as additional mass beyond the ash total. In the transport material definition, these cations were assigned to hypothetical CaO, MgO, K_2_O, and Na_2_O components, and the remaining ash was represented by a SiO_2_ proxy. This assignment is a bookkeeping approximation, not a mineralogical identification. The detailed cation inputs are supplied in Supplementary Data S1. Neutron transport depends on elemental/isotopic number densities and the corresponding energy-dependent microscopic cross sections; assigning the reported cations to oxide bookkeeping forms therefore affects the calculation primarily through the resulting elemental mass fractions, including the associated oxygen. The assumed oxide phases are not intended to represent experimentally identified mineralogy. Because the residual ash chemistry was not measured, this approximation remains a model-form uncertainty and is treated as such in the interpretation rather than as a characterization result.

### 2.2. Epoxy Matrix, Loading, Density, and Hydrogen Number Density

The primary matrix was an idealized effective epoxy composition, C_21_H_27_O_5_, with a nominal matrix density of 1.20 g cm^−3^. The reference composites contained 20 wt% biochar and 80 wt% epoxy. An effective biochar constituent density of 1.50 g cm^−3^ was assumed for the mixture-density estimate. The reciprocal rule of mixtures,

(1) $$\rho_{c}={\left(\frac{w_{m}}{\rho_{m}}+\frac{w_{b}}{\rho_{b}}\right)}^{-1},$$

gives 1.25 g cm^−3^ at 20 wt% biochar under these assumptions. The final comparison therefore used 1.25 g cm^−3^ for BC300, BC500, and BC800. The unfilled epoxy control (EP0) was also forced to 1.25 g cm^−3^ so that it served as a density-matched composition control; it should not be interpreted as the nominal density of the neat resin.

The value 1.50 g cm^−3^ is a controlled constituent-density assumption, not a measurement for the three Usman et al. biochars. Mahdi et al. reported a temperature-dependent sequence of particle-density numbers for date-seed biochar [12], summarized in a subsequent date-palm review [11]; however, the conference-paper table labels the density columns in g L^−1^ despite numerical magnitudes more commonly expressed in g cm^−3^. The original unit ambiguity and the different feedstock/temperature combinations preclude assigning those numbers to BC300, BC500, or BC800 or constructing a quantitatively reliable density-bound prediction from them. Actual constituent and cured-composite densities should be measured before extrapolating the equal-density chemistry comparison to manufactured materials.

For each modeled material, the hydrogen atom number density was calculated as

(2) $$n_{H}=\rho \;w_{H}\frac{N_{A}}{M_{H}},$$

where ρ is the composite density, $w_{H}$ is the hydrogen mass fraction, $N_{A}$ is Avogadro’s constant, and $M_{H}$ is the hydrogen molar mass. At 1.25 g cm^−3^, the resulting $n_{H}$ values are approximately 5.655, 5.133, 4.839, and $4.652\times 10^{22}$ atoms cm^−3^ for EP0, BC300, BC500, and BC800, respectively.

All materials were represented as homogeneous mixtures. Particle-size distributions, interfaces, voids, agglomeration, moisture, porosity, cure chemistry, and processing-induced density gradients were not modeled. The final inferential comparisons are therefore composition-controlled transport calculations rather than manufacturability predictions.

### 2.3. GEANT4 Transport Model

Neutron transport calculations were performed using GEANT4 10.6.2 [13,14] with the Shielding reference physics list and the G4NDL4.6 neutron data library. This computational configuration was maintained consistently throughout the simulation campaigns to ensure reproducibility and direct comparability of the results. The evaluated neutron cross-section data in G4NDL4.6 are derived mainly from JEFF-3.3 [15], while the Neutron-HP evaluated-data framework is discussed by Thulliez et al. [16]. No custom material-specific bound-atom thermal-scattering S(α,β) data were assigned to the epoxy or biochar materials.

The sample was a 10×10 cm^2^ slab with a baseline thickness of 5 cm. For the principal campaign, monoenergetic 2 MeV neutrons were emitted in the +z direction from points sampled uniformly in a disk of 2 cm radius located 2 cm upstream of the sample. The surrounding world was vacuum. A thin scoring volume of transverse area 100 cm^2^ and thickness 0.001 cm was positioned 5 mm beyond the downstream sample face. No downstream water phantom was present, preventing backscatter from an auxiliary scoring object from re-entering the tally region.

A second source condition represented ^252^Cf spontaneous-fission neutrons using the Watt form

(3) $$p\left(E\right)\propto \exp\left(-E/a\right)\sinh\;\left(\sqrt{bE}\right),$$

with a=1.025 MeV and b=2.926 MeV^−1^, parameters used in the SCALE/Monaco shielding benchmark documentation [17]. The distribution was sampled numerically over 0–20 MeV. This case is used as a representative broad fission-neutron sensitivity calculation rather than as a model of a particular encapsulated laboratory source.

### 2.4. Energy-Resolved Track-Length Fluence and $H^{*}\left(10\right)$ Weighting

The primary transport observable was a track-length fluence estimate in the thin scoring volume. For energy interval *j*, the forward component was calculated as

(4) $$\Phi_{j}=\frac{1}{NV}\sum_{s\in j,\;\mu_{z}>0}\ell_{s},$$

where *N* is the number of generated primary neutrons, *V* is the scoring-volume volume, $\ell_{s}$ is the neutron step length inside the scoring volume, and $\mu_{z}$ is the cosine of the neutron direction relative to the beam axis. The reported unit is cm^−2^ per generated primary. The plotted 2 MeV-source energy intervals were 0–0.01, 0.01–0.1, 0.1–0.5, 0.5–1.0, 1.0–1.5, 1.5–1.9, and 1.9–2.0 MeV. No ^252^Cf energy-bin histogram is presented: its component and total fluence and $H^{*}\left(10\right)$ integrals were accumulated over the full sampled 0–20 MeV source range, with transported-neutron energies weighted step by step, *without* an upper 2 MeV cutoff.

An $H^{*}\left(10\right)$-weighted response per primary was then calculated from the stepwise neutron fluence using the neutron fluence-to-ambient-dose-equivalent coefficients of ICRP Publication 74 and ICRU Report 57 [18,19], with the IAEA neutron-monitoring report used as an additional implementation reference [20]. Between tabulated energies, the coefficient was evaluated with four-point cubic Lagrange interpolation in log–log space. For the very small number of neutron steps below the lower tabulated energy of $10^{-9}$ MeV, the lowest tabulated coefficient (6.60 pSv cm^2^) was held constant rather than extrapolated. The complete 0–0.01 MeV interval contributes less than 0.4% of the total $H^{*}\left(10\right)$ response for every final material, so this lower-bound treatment cannot dominate the reported material differences. The resulting quantity is reported as an $H^{*}\left(10\right)$-weighted fluence response for the modeled forward field; it is not an experimentally measured ambient dose equivalent.

### 2.5. Scorer Verification, Component Classification, and Statistical Design

The track-length normalization was checked in vacuum using normally incident 2 MeV neutrons. For the original 100 cm^2^ tally, an uncollided primary crossing once should give 0.0100 cm^−2^ per primary; the calculation reproduced this value and the corresponding 4.20 pSv per-primary response obtained from the 2 MeV fluence-to-$H^{*}\left(10\right)$ coefficient. For the later 30×30 cm^2^ full-face sensitivity geometry, the analogous vacuum value was $1/900=1.1111\times 10^{-3}$ cm^−2^ per primary and was also reproduced. These are normalization checks rather than experimental validation.

The principal 2 MeV comparison used ten independent $10^{6}$-history replicates for EP0, BC300, BC500, and BC800. An independent component-classification campaign also used ten $10^{6}$-history replicates per material for both the 2 MeV and ^252^Cf source conditions. A primary neutron was classified as *uncollided* if it reached the scoring volume without a non-transport neutron interaction in the sample, as *collided primary* after one or more such interactions, and as *secondary* when its parent track ID was nonzero. The same track-length and $H^{*}\left(10\right)$ post-processing was applied to each class.

Replicate means are reported with SEMs. BC800–BC300 contrasts were treated as independent-group comparisons; 95% confidence intervals were calculated using the Welch standard error and Welch–Satterthwaite degrees of freedom. The ten-replicate component campaign replaced the preliminary three-replicate analysis used during manuscript development. Spectral-fraction uncertainties were calculated directly from the ten replicate-level energy-bin summaries.

Two additional sensitivity campaigns were performed after peer review. First, BC300 and BC800 were simulated in a 30×30 cm^2^, 5 cm-thick slab with a full-face 30×30 cm^2^ source and a correspondingly enlarged downstream tally, using ten independent $10^{6}$-history replicates per material. Second, one-factor material/geometry sensitivities compared BC300 and BC800 at densities of 1.15 and 1.35 g cm^−3^, slab thicknesses of 3 and 10 cm, and biochar loadings of 10 and 30 wt%, with five independent $10^{6}$-history replicates per material and condition. These calculations were designed to test robustness of the BC800–BC300 contrast, not to define an optimized engineering design.

Finally, the 2 MeV uncollided result was checked analytically against

(5) $$T_{\mathrm{unc}}=\exp\left(-\Sigma_{t}x\right),\;\Sigma_{t}=\rho N_{A}\sum_{i}\frac{w_{i}}{A_{i}}\sigma_{t,i},$$

where elemental total cross sections were reconstructed directly from the installed G4NDL4.6 elastic, inelastic, capture, and fission cross-section files at 2 MeV. Natural-isotope abundances were applied when a natural-element file was unavailable. Because this calculation uses the same evaluated-data family as the GEANT4 run, it is a material-definition and scorer-consistency verification rather than independent nuclear-data or experimental validation.

## 3. Results

### 3.1. Principal 2 MeV Comparison

Table 2 summarizes the principal ten-replicate campaign. BC800 exceeded BC300 by only $2.36\times 10^{-5}$ cm^−2^ primary^−1^ in total forward fluence (+0.30%), whereas the $H^{*}\left(10\right)$-weighted response increased from 2.6957 to 2.7729 pSv primary^−1^ (+2.86%). The BC500–BC300 integral-fluence difference was only +0.078% and its 95% interval included zero; therefore the data do not support a resolved monotonic increase in total fluence at every intermediate pyrolysis step.

The energy distribution changes more clearly than the integral fluence. The 1.9–2.0 MeV fraction increases from 43.736±0.015% for BC300 to 46.314±0.018% for BC800, a difference of 2.578 percentage points (95% CI 2.529–2.627). Conversely, the fraction below 0.1 MeV falls from 14.181±0.016% to 12.280±0.015%, a difference of −1.901 percentage points (95% CI −1.947 to −1.856). These changes are therefore much larger than their replicate-level Monte Carlo uncertainty.

**Figure 1:**
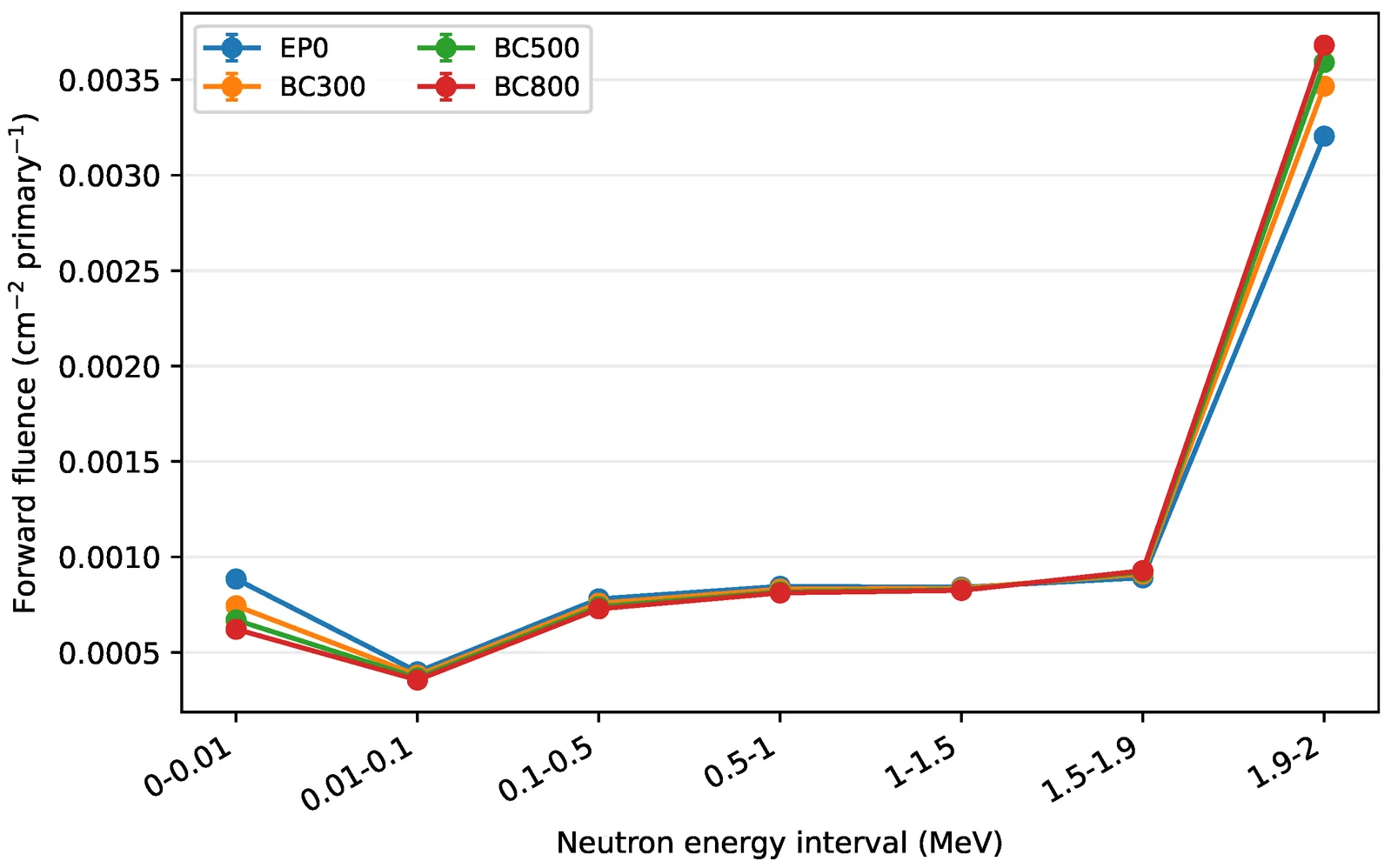
Energy-resolved forward neutron fluence for the density-matched epoxy control and the three 20 wt% biochar composites. Error bars are SEMs across ten independent replicates.

The energy-resolved fluence distributions are shown in Figure 1.

**Figure 2:**
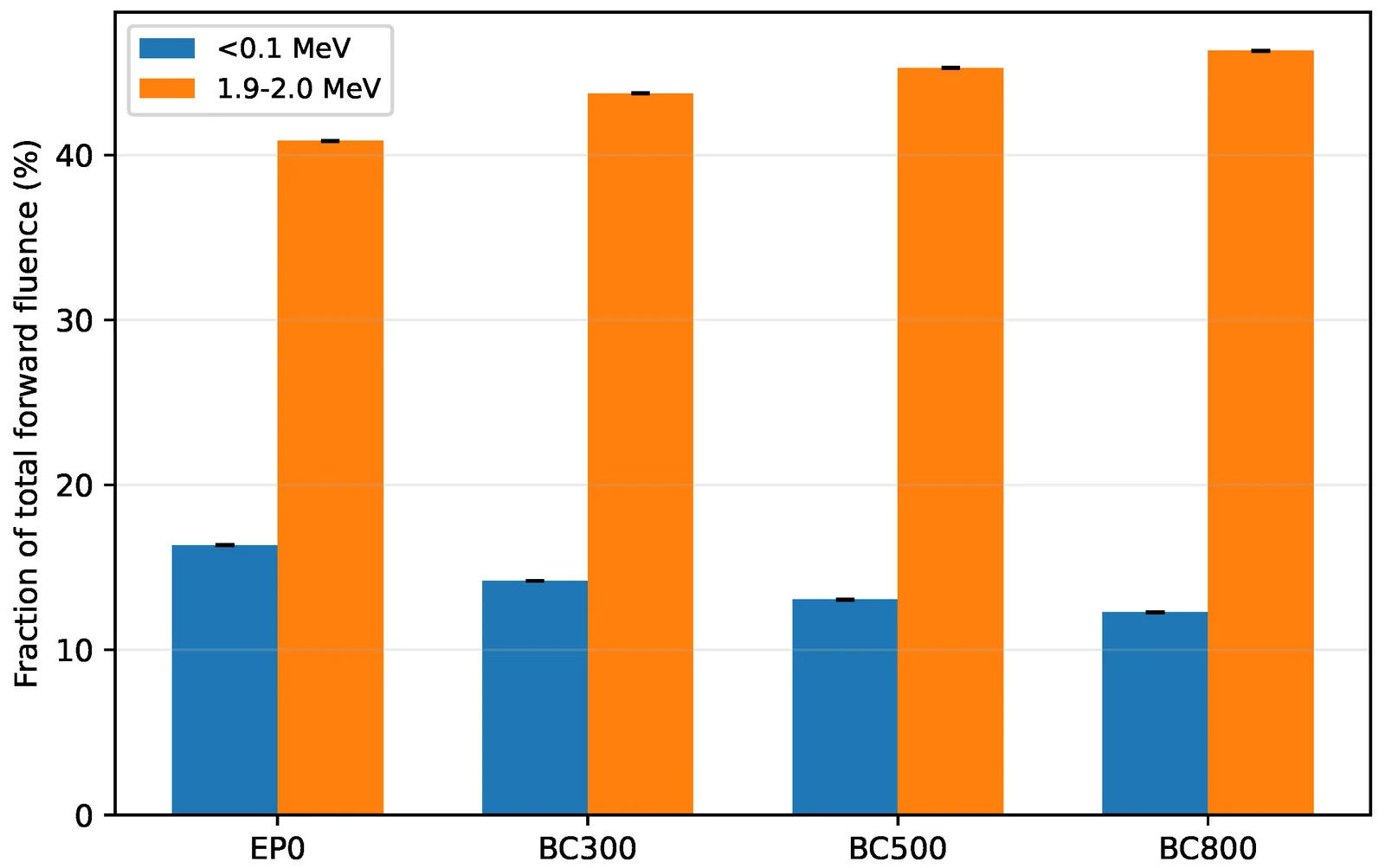
Low-energy (<0.1 MeV) and near-incident-energy (1.9–2.0 MeV) fractions of the total forward fluence. Error bars are SEMs across ten replicates.

Figure 2 compares the low-energy and near-incident-energy fractions of forward fluence.

### 3.2. Ten-Replicate Transport-Component Analysis

The independent component-classification campaign reproduced the principal ordering and identified its origin. At 2 MeV, BC300 and BC800 gave total $H^{*}\left(10\right)$ responses of 2.69398±0.00075 and 2.77384±0.00069 pSv primary^−1^, respectively (+2.96%). The BC800–BC300 difference was +0.07986 pSv primary^−1^ (95% CI 0.07772–0.08200). The uncollided-primary component increased by +0.08398 pSv primary^−1^ (95% CI 0.08224–0.08571), whereas the collided-primary component decreased by −0.00422 pSv primary^−1^ (95% CI −0.00634 to −0.00211). Thus, the net increase is generated by greater uncollided-primary survival, while the collided field slightly offsets it in the original narrow-beam configuration.

For ^252^Cf, total $H^{*}\left(10\right)$ increased from 2.26714±0.00065 to 2.33479±0.00068 pSv primary^−1^ (+2.98%). Of the +0.06765 pSv primary^−1^ difference, +0.06166 pSv primary^−1^ arose from uncollided primaries (91.1%), +0.00541 from collided primaries (8.0%), and +0.00059 from secondary neutrons (0.9%). All three component contrasts had Welch 95% intervals excluding zero.

**Figure 3:**
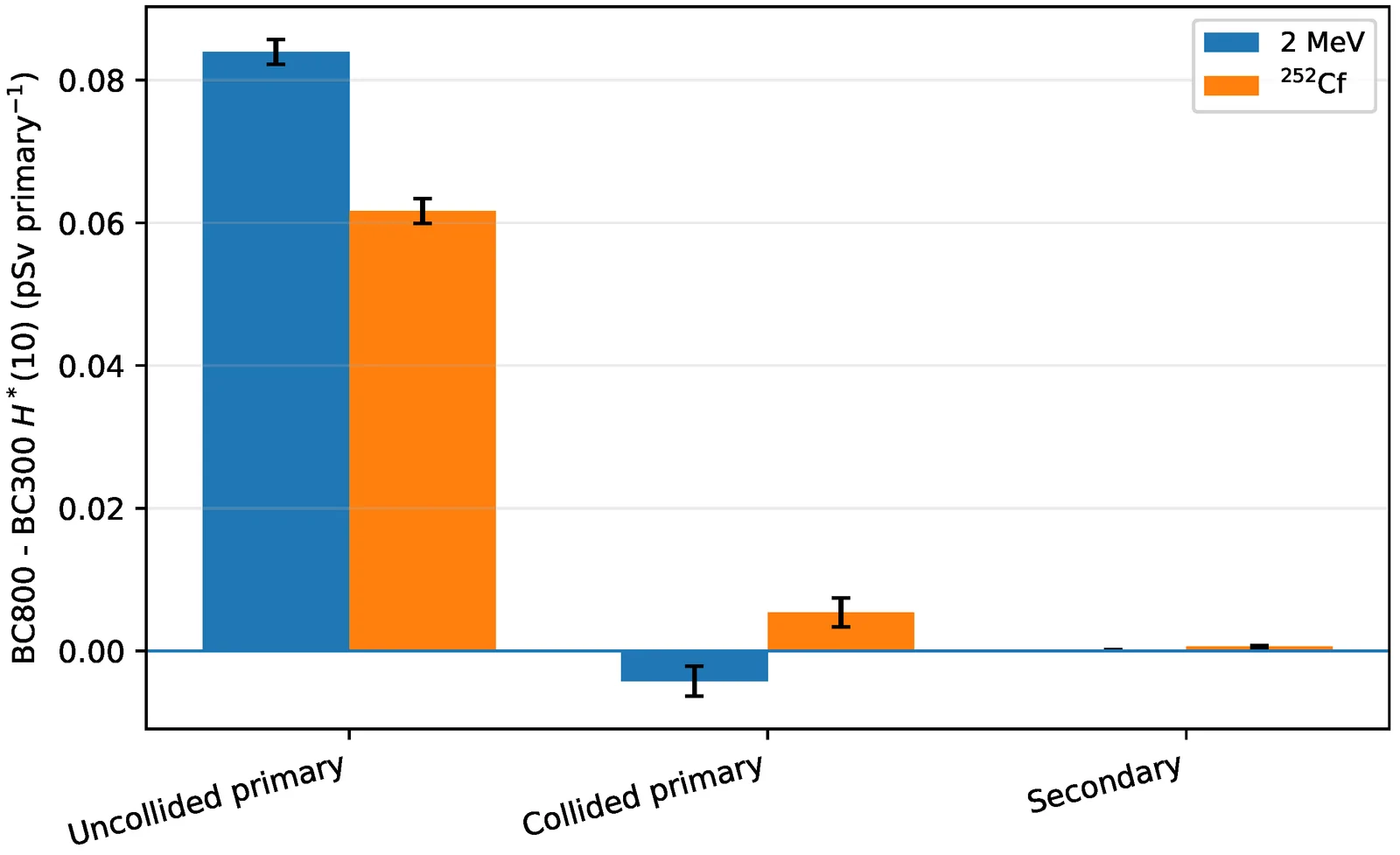
BC800–BC300 component differences in $H^{*}\left(10\right)$-weighted response for 2 MeV and ^252^Cf sources. Error bars are Welch 95% confidence intervals from ten independent replicates per material.

The component-resolved differences and their confidence intervals are summarized in Figure 3.

### 3.3. Evaluated-Data Consistency Check

The Beer–Lambert reconstruction gave 2 MeV macroscopic total cross sections of 0.254214, 0.238498, 0.231016, and 0.225753 cm^−1^ for EP0, BC300, BC500, and BC800. The corresponding Monte Carlo uncollided transmissions were systematically lower than the evaluated-data reconstruction by 0.43–0.51%. Because the signed offset exceeds the replicate sampling uncertainty, it is treated as a small systematic implementation difference rather than statistical noise. Plausible sources include natural-isotope mapping, table interpolation, and treatment of minor constituents, but the available records do not isolate a unique cause. Importantly, the offset is similar in sign and magnitude across all four materials and does not reverse their ordering. Hydrogen contributes approximately 64.6%, 62.5%, 60.8%, and 59.8% of reconstructed $\Sigma_{t}$ for EP0, BC300, BC500, and BC800, respectively.

### 3.4. Geometry Sensitivity: 30 × 30 cm Full-Face Irradiation

The larger geometry produced the expected increase in captured scattered fluence. Vacuum-normalized uncollided fluence remained nearly unchanged between geometries: 0.30220 (BC300) and 0.32177 (BC800) in the 30 cm full-face case, compared with approximately 0.302 and 0.322 in the original geometry. In contrast, normalized collided fluence increased to 0.5830 for BC300 and 0.5687 for BC800 because lateral escape was reduced.

Despite this change in scattered buildup, BC800 still exceeded BC300 in total $H^{*}\left(10\right)$ by 3.47% (0.324942±0.000141 versus 0.314060±0.000087 pSv primary^−1^). Of the +0.010882 pSv primary^−1^ difference, 84.0% arose from uncollided primaries, 16.0% from collided primaries, and less than 0.1% from secondaries. The collided contrast therefore changes sign between the narrow-beam and full-face geometries, confirming that the scattered-field partition is geometry-dependent; however, increased uncollided-primary survival remains the dominant BC800–BC300 mechanism in both cases.

**Figure 4:**
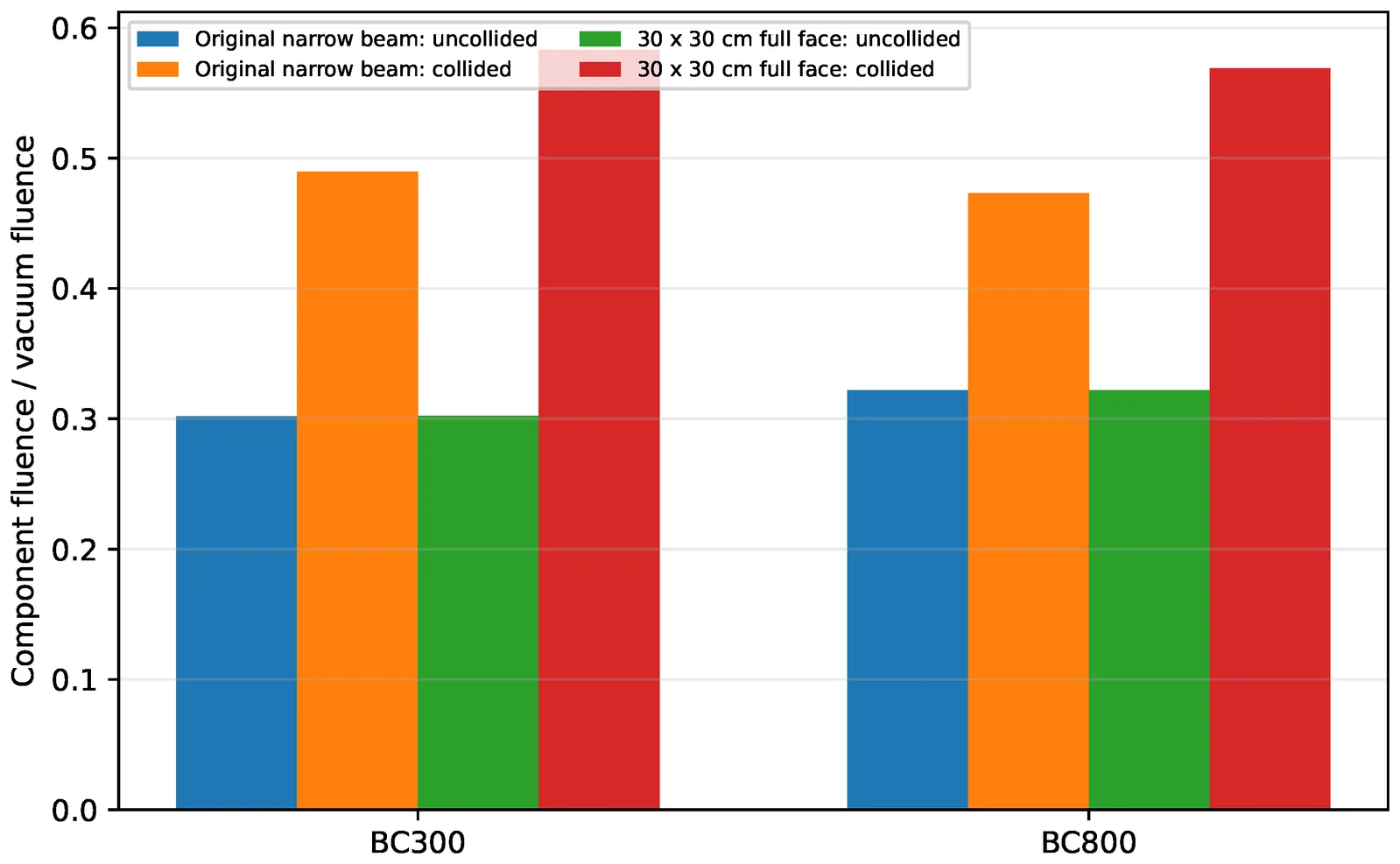
Vacuum-normalized uncollided and collided forward fluence for BC300 and BC800 in the original narrow-beam configuration and the 30×30 cm^2^ full-face sensitivity geometry.

Figure 4 compares uncollided and collided contributions between the two scoring geometries.

### 3.5. Sensitivity to Density, Thickness, and Filler Loading

One-factor sensitivity calculations show that the absolute response is strongly parameter-dependent, especially with respect to thickness. Nevertheless, the BC800–BC300 $H^{*}\left(10\right)$ contrast remained positive for every tested condition: +2.53% at 1.15 g cm^−3^, +3.35% at 1.35 g cm^−3^, +1.18% at 3 cm, +8.24% at 10 cm, +1.51% at 10 wt% biochar, and +4.19% at 30 wt% biochar. The corresponding Welch 95% intervals on the absolute $H^{*}\left(10\right)$ differences excluded zero in all six cases.

The total-fluence contrast was less robust. At 3 cm thickness, BC800 produced 0.295% lower total forward fluence than BC300 while still giving a 1.18% higher $H^{*}\left(10\right)$ response. This result reinforces that the principal pyrolysis-dependent signal is energy-dependent transmission and dose weighting rather than a universal monotonic increase in integral fluence. Across all six parameter sensitivities, the positive uncollided-primary contribution remained the largest contributor to the BC800–BC300 $H^{*}\left(10\right)$ difference, whereas the collided term varied in both magnitude and sign.

**Figure 5:**
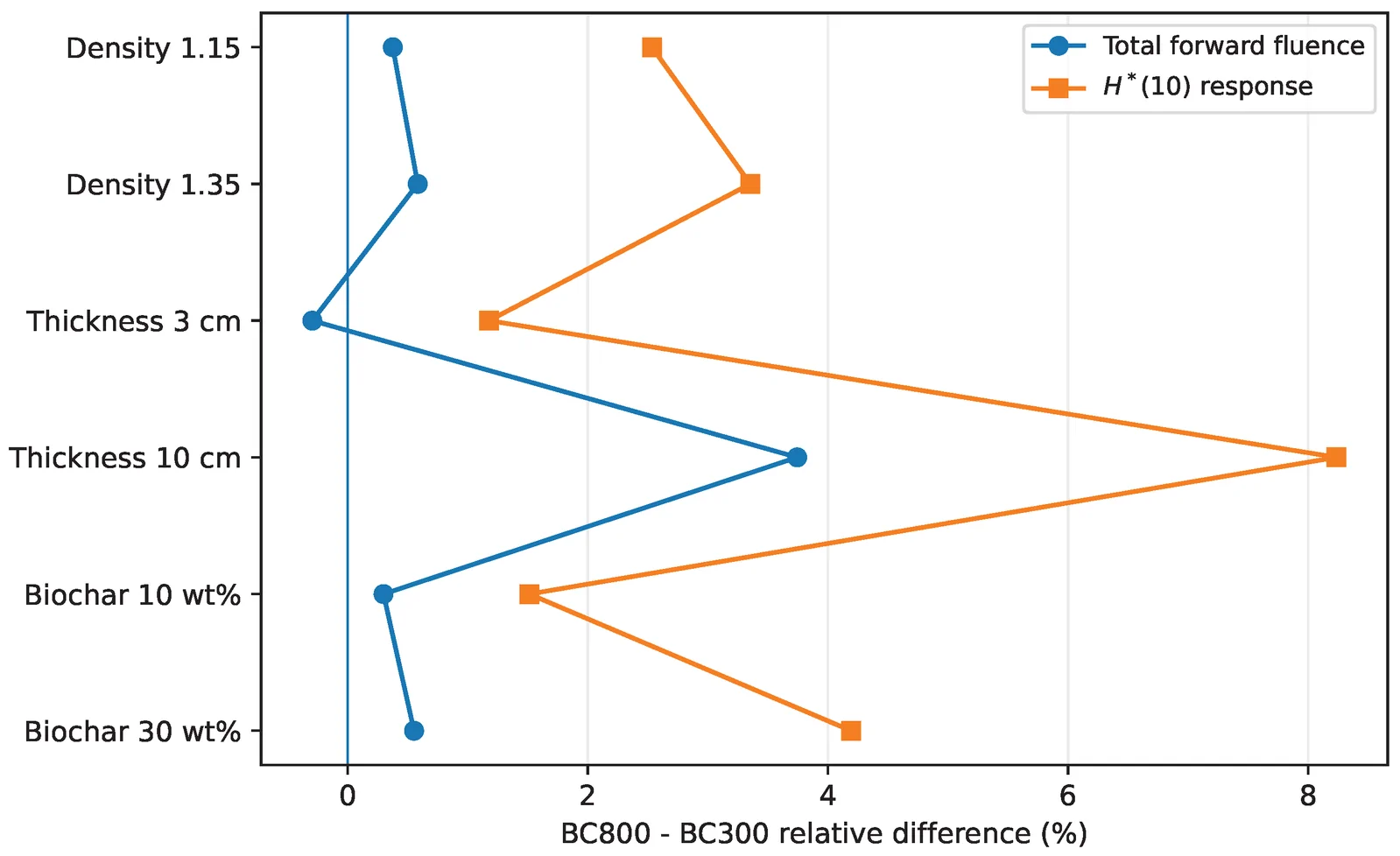
Relative BC800–BC300 differences in total forward fluence and $H^{*}\left(10\right)$ response for one-factor density, thickness, and biochar-loading sensitivities. Each sensitivity condition used five independent $10^{6}$-history replicates per material.

Figure 5 summarizes the relative total-fluence and $H^{*}\left(10\right)$ contrasts across the sensitivity cases.

## 4. Discussion

### 4.1. Mechanistic Interpretation and Practical Significance

The revised calculations separate two conclusions that should not be conflated. First, the integral forward fluence changes only modestly with pyrolysis chemistry and is not monotonic under every sensitivity condition. Second, the energy distribution and $H^{*}\left(10\right)$-weighted response show a more persistent BC300–BC800 difference. This distinction is physically consistent with the decreasing hydrogen number density from BC300 to BC800: reduced interaction probability allows a larger fraction of higher-energy primaries to survive the slab, which receives greater weight in the neutron fluence-to-$H^{*}\left(10\right)$ conversion.

The ten-replicate component analysis provides direct evidence for this interpretation. In the original geometry, the uncollided component is larger than the total BC800–BC300 increase because the collided field partly compensates in the opposite direction. In the larger full-face geometry, the collided contribution becomes positive because more scattered neutrons are retained, yet 84% of the net $H^{*}\left(10\right)$ difference still comes from uncollided primaries. The sign of the scattered contrast is therefore geometry-dependent, whereas increased uncollided survival is comparatively robust. The analytic G4NDL reconstruction independently reproduces the material ordering within the same nuclear-data family and supports the interpretation that hydrogen depletion is the dominant first-order driver.

The ^252^Cf calculation further shows that the mechanism is not restricted to a monoenergetic 2 MeV beam. The fission-spectrum result preserves an approximately 3% BC800–BC300 $H^{*}\left(10\right)$ difference, with more than 90% of that increase arising from uncollided primaries in the original geometry. This does not establish performance for every practical source, but it broadens the energy context beyond a single incident energy.

### 4.2. Relation to Polymer-Shielding Literature and Design Use

Prior experimental polymer shields commonly combine a hydrogen-rich phase with a neutron absorber such as B_4_C [6,7,8]. Those systems seek high moderation and absorption together, whereas the present biochar/epoxy study asks a different screening question: how does replacing part of the hydrogen-bearing matrix with a pyrolysis-dependent biomass-derived filler alter transport before any dedicated absorber is introduced? The result is therefore best interpreted as a compositional-screening constraint for multifunctional composite design rather than a claim that neat biochar should replace conventional neutron-shielding fillers.

Biochar may still be attractive for renewable-carbon content, waste valorization, stiffness, electrical conductivity, thermal response, cost, or other multifunctional objectives [1,2,3,4]. For radiation-sensitive applications, the present model indicates that higher-temperature biochar can modestly increase the survival of high-energy neutrons relative to a lower-temperature biochar at equal density. This information can be used during pre-fabrication screening to balance radiation transport against mechanical, thermal, processing, and sustainability requirements. Potential beneficiaries include laboratory and industrial shielding components, accelerator and nuclear-technology systems, radiation facilities, and lightweight protective structures, provided that application-specific source spectra and geometry are modeled and experimentally verified.

### 4.3. Limitations and Validation Status

The present study employs a controlled, density-matched material model to isolate the influence of pyrolysis-dependent biochar composition on neutron transport. The epoxy matrix is represented by an idealized effective chemical formula, while the biochar constituents are modeled as homogeneous elemental mixtures. The residual ash mineralogy is approximated using oxide-based elemental bookkeeping. These assumptions provide a consistent basis for comparing BC300, BC500, and BC800 but do not reproduce all the structural and chemical characteristics of fabricated composites. In particular, porosity, particle morphology, moisture, filler dispersion, and interfacial effects are not explicitly modeled. The additional density, thickness, and filler-loading sensitivity calculations help quantify the dependence of the predicted response on selected material and geometric parameters.

The original finite-slab, narrow-beam configuration was supplemented by a 30×30 cm^2^ full-face geometry calculation. The results confirm that scattered-neutron buildup and the collided-primary contribution depend on lateral dimensions and source/tally acceptance. Nevertheless, the increased survival of uncollided primary neutrons remains the dominant contribution to the BC800–BC300 difference in the $H^{*}\left(10\right)$-weighted response for both geometries. The calculated observables represent forward neutron fluence and its $H^{*}\left(10\right)$ weighting under the specified simulation conditions, rather than experimentally measured shielding performance.

The evaluated-data attenuation calculation provides an additional consistency check, reproducing the GEANT4 uncollided transmission within approximately 0.5%. Because this reconstruction uses the same underlying evaluated nuclear-data family, it is not an independent experimental validation. Experimental confirmation would require fabricated and characterized biochar/epoxy specimens, measured composite density and composition, and neutron measurements under a corresponding source–sample–detector configuration.

## 5. Conclusions

A composition-resolved GEANT4 study was performed for idealized date-palm biochar/epoxy composites to determine which pyrolysis-dependent neutron-transport effects are robust and which depend on geometry or material assumptions. In the principal 20 wt%, 5 cm, equal-density comparison, BC800 exceeded BC300 by only 0.30% in total forward fluence but by 2.86% in the $H^{*}\left(10\right)$-weighted response. Ten-replicate component calculations showed that this difference is driven by greater survival of uncollided primary neutrons; at 2 MeV the uncollided contribution increased by 0.08398 pSv primary^−1^ while the collided component decreased slightly. For a representative ^252^Cf spectrum, $H^{*}\left(10\right)$ increased by 2.98%, with 91.1% of the net increase arising from uncollided primaries.

A 30×30 cm^2^ full-face sensitivity increased scattered buildup and reversed the sign of the collided BC800–BC300 contrast, demonstrating that the scattered field is geometry-dependent. Even so, uncollided primaries still accounted for 84.0% of the total $H^{*}\left(10\right)$ increase. Density, thickness, and filler-loading sensitivities likewise retained a positive BC800–BC300 $H^{*}\left(10\right)$ contrast under all tested conditions (1.18–8.24%), whereas the total-fluence difference was not universally positive. The robust conclusion is therefore not that increasing pyrolysis temperature always raises integral neutron transmission, but that hydrogen depletion increases high-energy primary survival and can increase dose-weighted downstream response under the modeled conditions.

These results provide a computational screening framework for epoxy/biochar formulations, not a fabrication prescription. Real composite performance will depend on measured density, porosity, moisture, particle morphology, dispersion, ash mineralogy, source spectrum, and shielding geometry. Experimental validation with fabricated and compositionally characterized specimens remains necessary before the calculations are interpreted as engineering shielding performance.

## Figures and Tables

**Table 1:** Date-palm biochar input composition (wt%) used in the model. The values are from Usman et al. [10]. Cation concentrations used in the ash bookkeeping are provided in Supplementary Data S1.

Sample	C	H	O	N	S	Ash
BC300	57.99	4.08	20.82	0.54	2.14	14.42
BC500	72.30	2.11	4.50	0.42	1.02	19.68
BC800	74.63	0.86	2.27	0.31	0.54	21.39

**Table 2:** Principal 2 MeV results. Values are means ± SEM across ten independent $10^{6}$-history replicates. EP0 is a density-matched composition control.

Material	$n_{H}$	Forward Fluence	$H^{*}\left(10\right)$	<0.1 MeV	1.9–2.0 MeV
	( $10^{22}$ cm^−3^)	(cm^−2^ primary^−1^)	(pSv primary^−1^)	(% fluence)	(% fluence)
EP0	5.655	0.0078429±0.0000028	2.5914±0.0009	16.340±0.019	40.853±0.026
BC300	5.133	0.0079254±0.0000026	2.6957±0.0010	14.181±0.016	43.736±0.015
BC500	4.839	0.0079316±0.0000029	2.7389±0.0011	13.054±0.020	45.272±0.024
BC800	4.652	0.0079490±0.0000024	2.7729±0.0007	12.280±0.015	46.314±0.018

## Data Availability

All processed numerical data required to reproduce the reported tables and figures, the component-resolved summaries, the evaluated-data consistency check are provided in the Supplementary Materials. The date-palm biochar composition originates from the published dataset of Usman et al. [10].

## Supplementary Materials

The following supporting information can be downloaded at https://www.mdpi.com/article/10.3390/polym18212687/s1, Supplementary Data S1: Date-palm biochar elemental composition and ash-cation inputs; Supplementary Data S2: Principal ten-replicate 2 MeV results and energy-bin summaries; Supplementary Data S3: Transport-component results and confidence intervals; Supplementary Data S4: Evaluated-data uncollided-transmission consistency check; Supplementary Data S5: Full-face geometry sensitivity results; Supplementary Data S6: Density, thickness, and filler-loading sensitivity results.
